# The Pathophysiology of Impulse control Disorders in Parkinson Disease

**DOI:** 10.3233/BEN-2012-120266

**Published:** 2013

**Authors:** Sharmili Balarajah, Andrea Eugenio Cavanna

**Affiliations:** ^1^Department of NeuropsychiatryThe Michael Trimble Neuropsychiatry Research GroupBSMHFT and University of BirminghamBirminghamUK; ^2^Department of Motor Neuroscience and Movement DisordersInstitute of Neurology and University College LondonLondonUK

**Keywords:** Deep brain stimulation, dopamine agonists, impulse control disorders, levodopa, Parkinson's disease, pathological gambling

## Abstract

*Aims:* This review aims to evaluate the most recent evidence on the pathophysiology of impulse control disorders (ICDs) in Parkinson disease (PD).

*Methods:* Computerised searches of Medline, Embase and PsycInfo, along with manual searches for grey literature, were conducted and resulted in a total of 16 studies suitable for review.

*Results:* Evidence was divided into four categories: medication used in PD management, imaging studies, genetic analysis and subthalamic deep brain stimulation (STN-DBS). Analysis of the literature reveals that both intrinsic and extrinsic factors may play a role in the pathophysiology of ICDs in PD. Dysfunction of the mesocorticolimbic pathway and polymorphisms of the dopamine D3 and D4 receptors may increase an individual's susceptibility to the development of ICDs.

*Discussion:* Dopaminergic medication, particularly dopamine agonists (DAs), increases the risk of developing impulsive behaviours in a PD patient. Further evidence, particularly in the form of prospective studies and randomised controlled trials is required to better establish the pathophysiology of ICDs in PD.

## 1. Introduction

Parkinson disease (PD) is a multisystem neurological condition estimated to affect 6–11 per 6,000 of the general UK population [1]. PD is characterised by the depletion of dopaminergic neurons in the substantia nigra, resulting in an imbalance of the basal ganglia circuitry. This depletion of dopaminergic neurons instigates a variety of symptoms, which can be broadly categorised into motor and non-motor. Whilst much research has provided an insight into the pathophysiology of motor symptoms in PD, this is less so the case for non-motor symptoms.

Studies have demonstrated a neuropsychiatric element of PD, one of the most common being impulse control disorders (ICDs) [2]. ICDs is an umbrella term covering a spectrum of conditions, which include pathological gambling and compulsive behaviours such as binge eating and hypersexuality [3] and is defined by the Diagnostic and Statistical Manual of Mental Disorders (DSM-IV-TR) as a failure to resist an impulse, drive or temptation to perform an act that is either physically, psychologically, socially, legally or financially harmful to the patient or others [4]. The prevalence of ICDs in PD varies from 1.7–6.1% for gambling to 0.4–3% for hypersexuality and their significance is highlighted by the mention of ICDs in the National Institute for Clinical Excellence (NICE) guidelines for PD [1].

Our current understanding of reward and addiction mechanisms predominantly involves dopaminergic mesocorticolimbic pathways, which encompass sever-

ISSN 0953-4180/13/$27.50 © 2013 – IOS Press and the authors. All rights reserved al brain regions including the prefrontal cortex, ventral striatum and amygdala [3]. Considering pathophysiology of PD, there is evidence that the depletion of dopaminergic neurons not only affects the nigrostriatal pathway but the mesocorticolimbic pathway too [5]. Dopaminergic medication may also induce hyperactivity of this pathway given the presence of several subtypes of dopaminergic receptors within it [6].

Although the symptoms of ICDs can be extremely disabling [7,8], the evidence base for their treatment is limited [9,10], and therefore there is a need to gain further understanding of the pathophysiology of ICDs in PD. This review will evaluate the most recent evidence on the pathophysiology of ICDs in PD.

## 2. Methods

We conducted a systematic literature review according to the methodology suggested by the Prisma guidelines. Computerised searches were run using PubMed, Medline, PsycInfo, Embase, the Cochrane library and Google Scholar. The following search strategy was used: (*addict OR gambl* OR impulse OR reward OR dopamine dysregulation syndrome OR compuls* OR hypersexual**) *AND Parkinson**. In addition to references obtained by searching the databases mentioned above, the reference lists of pertinent articles were scanned as were the contents pages of significant journals that may have a greater focus on neuropsychiatry and PD (Brain, Journal of Neuropsychiatry and Clinical Neurosciences, The Lancet Neurology, Movement Disorders, Nature Neuroscience, Parkinsonism and Related Disorders).

### 2.1. Study inclusion criteria

To obtain an idea of the current understanding of the pathophysiology of ICDs in PD, references were limited to human studies published within the last three years (2008–July 2011). Of these studies, those to be included in this review were required to have a sample size of greater than $n = 20$.

### 2.2. Study exclusion criteria

Other types of articles, such as case reports, case series, letters, editorials and reviews, were excluded from this review. Non-English articles were also excluded.

Once duplicates had been removed from the search results, titles and abstracts were initially reviewed to identify potentially eligible studies. Reference titles and abstracts were scanned and excluded if deemed unfitting to the search topic. The full text of studies that had not been excluded at this stage was then assessed to determine eligibility for inclusion in this review.

## 3. Results

### 3.1. Overview of search results

A total of 268 potentially eligible articles were identified through literature searches (264 from electronic searches and 4 from review of the grey literature). Of these articles 16 were suitable for inclusion in this review.

### 3.2. Influence of PD medication in development of ICDs (Table 1)

Five recent studies have confirmed that the use of medications in the management of PD can lead to the development of ICDs in patients. Pramipexole, one of the most commonly used dopamine agonists (DAs) has been positively associated with patients with PD who have developed ICDs [24,25] and has been shown to increase the risk of developing PG by 3.65-fold [25]. Positive correlations found between the total daily levodopa equivalent dose (LED) of DAs and impulsivity [26] provide a strong indication of the involvement of DAs in the development of ICDs in PD. Levodopa, on the other hand, appears to possess a less influential role in the development of ICDs compared to DAs as shown by Weintraub et al. [28]. Evidence suggests, however, that there may be also be an underlying neurobiological vulnerability, which influences ICD development in patients with PD [27].

### 3.3. Imaging studies (Table 2)

Imaging studies use a range of techniques including functional magnetic resonance imaging (fMRI), positron emission tomography (PET) and voxel-based morphometry (VBM) to investigate the neurobiological correlates of ICDs in PD. Several studies have demonstrated higher rates of activation of the prefrontal cortex, particularly within the orbitofrontal cortex (OFC) [30,31,33]. These studies also implicate the involvement of the striatum in impulsive behaviours: increased striatal activity has been found in both PD patients with ICDs [31,33,34] and PD controls who are

Table 1
Pharmacotherapy for Parkinson disease and impulse control disorders

| Study | Design | Participants | Inclusion criteria | Methodology | Findings |
|---|---|---|---|---|---|
| Ondo and Lai, 2008 [24] | Cross-sectional | 300 patients on DA medication (207 PD, 89 RLS, 4 PD & RLS) | Not stated | Clinical assessment of gambling habits, spending behaviour, sexual behaviour/desire and open miscellaneous | – Impulsive behaviour more common in DA agonist-medicated PD patients vs. RLS patients<br>– Higher dose of DA agonist found to be a significant risk factor for the development of reward seeking behaviour |
| Imamura et al., 2008 [25] | Case-control | 48 (11 PD patients with newly developed PG & 37 age, sex-matched PD controls) | Diagnosis of PD by neurologist | | – Greater use of pramipexole in cases (PD patients with PG) than controls (PD patients without PG<br>– Insignificant difference in LED between cases and controls but significant but significant difference in dose of pramipexole (3.65-fold risk of patients developing PG if on pramipexole) |
| Torta et al., 2009 [26] | Case-control | 28 (15 PD patients on and off medication & 13 healthy controls) | Evaluation by two neurologists | CGT | – Significantly greater impulsivity in PD patients (on and off medication) vs. controls<br>– Positive correlation between degree of impulsivity and LED |
| Voon et al., 2010a [27] | Case-control | 42 (14 PD patients with ICDs, 14 PD patient controls, 16 medication-free matched controls) | PD – Queen Square Brain Bank diagnostic criteria<br>ICDs – DSM-IV-TR/McElroy's criteria | EDT | – DA associated with increasing impulsivity in PD patients with ICDs but not controls<br>– PD patients with ICDs on DA tend to overestimate a risky choice compared to off |
| Weintraub et al., 2010 [28] | Cross-sectional | 3090 idiopathic PD patients | Not stated (recruited from movement disorder centres in Canada and America) | – Massachusetts Gambling Screen<br>– MIDI<br>– DSM-IV-TR | – Significantly higher frequency of ICDs found in DA-treated PD patients<br>– 2.6-fold risk of ICD development in patients treated with DA than with levodopa<br>– Combination treatment with levodopa and DA provides greater risk of development of ICDs than DA alone |

CGT Cambridge gambling task; DA dopamine agonist; DSM-IV-TR Diagnostic and Statistical Manual of Mental Disorders, 4th edition, text revision; EDT experimental discounting task; ICDs impulse control disorders; LED total daily levodopa daily equivalent dose; MIDI Minnesota Impulsive Disorders Interview; PD Parkinson disease; PG pathological gambling; RLS restless legs syndrome.

taking DA [33]. Involvement of the insular cortex has been implied after imaging has shown an increased activation in patients with PD who demonstrate impulsive behaviours [31]. VBM has demonstrated anatomical change with a loss of volume of both the amygdala and OFC found in patients with ICDs compared to healthy controls [32].

### 3.4. Genetic influence on development of impulsive behaviour (*Table 3*)

Polymorphism of the D3 dopamine receptor is independently associated with the development of impulsive behaviours in patients with PD. No such association has been found for other variants of dopamine receptors [35]. Eisenegger et al. [36] has also demonstrated a further possible genetic component that may influence the development of compulsive behaviours in patients with PD: administration of L-DOPA was found to result in significantly greater gambling tendencies in healthy males with the dopamine receptor 4/7 polymorphism genotype compared to the 4/4 polymorphism.

### 3.5. Surgical studies (*Table 4*)

Subthalamic nucleus (STN) involvement in the development of ICDs in patients with PD has been sug-

Table 2
Imaging studies of impulse control disorders in Parkinson disease

| Study | Design | Participants | Inclusion criteria | Methodology | Findings |
|---|---|---|---|---|---|
| Hollander et al., 2008 [29] | RCT (double-blind) | 53 (21 PD PG patients [lithium or placebo], 32 age and sex-matched controls) | Structured clinical interview for DSM-IV with diagnosis of PG | FDG-PET scan | – rGMR within orbitofrontal lobe of prefrontal cortex greater in PD PG patients than in controls<br>– PG patients demonstrated reduced grey matter activity compared to controls at baseline |
| Keitz et al., 2008 [30] | Case-control | 23 (11 PD patients and 12 healthy controls) | UK Parkinson's Disease Society Brain Bank criteria | fMRI with monetary feedback | – Significantly increased activity in medial prefrontal cortex with monetary feedback in PD patients vs. controls |
| Cilia et al., 2008 [31] | Case-control | 80 (11 PD patients with PG, 40 matched PD controls, 29 age-matched healthy controls) | DSM-IV-TR (PG) | SPECT | – PD patients with PG vs. PD controls<br>∗ Increased clusters of perfusion in right lateral OFC extending into insula and globus pallidus with extension into nucleus accumbens<br>∗ Greater rCBF in right hippocampus, parahippocampal gyrus and amygdala<br>– PD patients with PG vs. healthy controls<br>∗ Increased activity in insular cortex, lateral OFC with involvement of putamen and caudate nucleus |
| Ibarretxe-Bilbao et al., 2009 [32] | Case-control | 48 (24 PD and 24 age, gender and education-matched controls) | UK Parkinson's Disease Society Brain Bank criteria | IGT; VBM | – PD patients had an increased tendency to select less advantageous decks in the IGT than controls<br>– VBM demonstrated significant loss of volume in right amygdala and bilaterally in orbitofrontal cortex |
| Voon et al., 2010b [33] | Case-control | 44 (14 PD patients with ICDs, 14 PD control patients and 16 healthy controls) | Queen Square Brain Bank criteria; DSM-IV-TR and McElroy's criteria (ICDs) | Probabilistic learning task; fMRI | – PD patients with ICDs – enhanced learning from gain outcomes (also associated with increased ventral striatal activity); greater OFC activity vs. PD controls in response to gain and loss<br>– PD controls taking DA demonstrated slower learning to negative outcomes and demonstrated increased activity in the anterior insular and right orbitofrontal cortices |
| O'Sullivan et al., 2011 [34] | Case-control | 18 L-DOPA treated PD patients (11 with ICDs, 7 without) | Queen Square Brain Bank for Neurological Disorders | $^{11}$C-raclopride PET scan; reward-related cues | – Exposure to reward-related cues demonstrated positive correlation between sensation seeking and $^{11}$C-raclopride binding potential in putamen and caudate nuclei in PD patients with ICDs<br>– $^{11}$C-raclopride binding potential in ventral striatum significantly reduced in PD patients with ICDs vs. PD controls post-reward cue exposure |

DA dopamine agonist; DSM-IV-TR Diagnostic and Statistical Manual of Mental Disorders, 4th edition, text revision; FDG-PET 18F-fluorodeoxyglucose positron emission tomography; fMRI functional magnetic resonance imaging; ICDs impulse control disorders; IGT Iowa gambling task; L-DOPA levodopa; OFC orbitofrontal cortex; PD Parkinson disease; PG pathological gambling; rCBF regional cerebral blood flow; RCT randomised controlled trial; rGMR relative glucose metabolic rates; SPECT single photon emission computed tomography; VBM voxel-based morphometry.

Table 3
Genetic influence on the development of impulse control disorders in Parkinson disease

| Study | Design | Participants | Inclusion criteria | Methodology | Findings |
|---|---|---|---|---|---|
| Lee et al., 2009 [35] | Cross-sectional | 963 (404 PD patients, 559 healthy controls) | UK Parkinson's Disease Society Brain Bank diagnostic criteria; MIDI | – Genotyping for allelic variants of dopamine and glutamate receptors; serotonin transporter genes | – DRD3 p.S9G polymorphism independently associated with impulsive behaviour in PD patient group<br>– No association found with DRD2Taq1A polymorphism |
| Eisenegger et al., 2010 [36] | RCT (double-blind, placebo controlled) | 200 males | Exclusion of significant medical disorders (particularly psychiatric and neurological) | – L-DOPA vs. placebo administration followed by gambling task | – Subjects with dopamine receptor 4/7 polymorphism had increased gambling tendency vs. 4/4 polymorphism |

MIDI Minnesota Impulsive Disorders Interview; PD Parkinson disease; RCT randomised controlled trial.

Table 4
Deep brain stimulation and impulse control disorders in Parkinson disease

| Study | Design | Participants | Inclusion criteria | Methodology | Findings |
|---|---|---|---|---|---|
| Hälbig et al., 2009 [37] | Cross-sectional | 53 (16 PD + DBS patients and 37 PD controls) | UK Parkinson's Disease Society Brain Bank diagnostic criteria | BIS | – BIS scores significantly higher in PD patients receiving DBS treatment vs. those without |
| Rodriguez-Oroz et al., 2011 [38] | Case-control | 28 patients with surgically implanted STN (10 PD patients with ICDs, 9 patients with L-DOPA-associated dyskinesia, 9 PD controls) | UK Parkinson's Disease Society Brain Bank diagnostic criteria | EEG recordings in 'on' and 'off' motor states | – Specific oscillatory activity in theta-alpha band identified in subthalamic nucleus region in PD patients with ICDs<br>– L-DOPA induces theta-alpha band oscillatory activity in the ventral subthalamic nucleus in PD patients with ICDs |
| Oyama et al., 2011 [39] | Clinical trial | 32 (16 PD patients for STN-DBS and 16 age-matched PD controls on dopaminergic medication) | UK Parkinson's Disease Society Brain Bank diagnostic criteria | IGT (pre-op and 2–4 weeks post-op) | – IGT demonstrated increased impulsivity in patients on STN-DBS vs. PD controls<br>– Poorer performance in IGT by STN-DBS patients during 'on' session than 'off' |

BIS Barratt impulsiveness scale; DBS deep brain stimulation; EEG electroencephalography; ICDs impulse control disorders; IGT Iowa gambling task; L-DOPA levodopa; PD Parkinson disease; STN-DBS subthalamic nucleus – deep brain stimulation.

gested by several recent studies [37–39]. These studies demonstrate that patients receiving subthalamic nucleus deep brain stimulation (STN-DBS) show a higher prevalence of impulsive behaviours than controls [37, 39]. STN involvement is also proposed given that L-DOPA induces significant oscillatory activity in this region in affected patients [38].

## 4. Discussion

Of the 16 original studies which have been reviewed to appraise the current evidence on the pathophysiology of ICDs in PD 31.2% focussed on the medications used in the management of PD, 37.5% on imaging studies undertaken at baseline and during tasks to induce impulsive activity, 12.5% on genetic predisposition and 18.8% on the outcome of STN-DBS.

DAs can be seen as one of the main culprits in the induction of impulsive behaviour in DA-medicated patients, with potential to increase the likelihood of ICD development by 3 times [28]. The potential to induce compulsive behaviour in patients with PD whilst improving motor symptoms may be due to underlying involvement of dopamine receptor subtypes. Pramipexole, one of the most commonly used DAs, has been shown to be relatively selective to dopamine D3 receptors as opposed to D1 and D2 receptors [11]. Evidence suggests uneven distribution of these dopamine receptor subtypes within different brain regions: D1 and D2 receptors are found to be abundant in the dorsal striatum whilst D3 receptors are found in abundance in the ventral striatum [12]. Whilst action of dopamine within the dorsal striatum may improve motor symptoms [12], activation of D3 receptors of the ventral striatum may induce impulsive behaviour [13,14].

The involvement of dopamine receptors has also been suggested by genetic studies such as that of Eisenegger et al. [36], who demonstrated a genetic predisposition to pathological gambling in individuals with a particular polymorphism of the dopamine D4 receptor. Dopamine D4 receptors are located within the mesocorticolimbic pathway, particularly within the nucleus accumbens [15]. It may be that the 4/7 polymorphism of the dopamine D4 receptor is more susceptible to dopamine-mediated activation than the 4/4 polymorphism, leading to overactivity of the mesocorticolimbic pathway and, therefore, an increased tendency towards impulsive behaviours. Dopamine D3 receptors, predominantly distributed within the limbic system [6,16] regulate dopamine release within the mesocorticolimbic pathway [17]. The association of the DRD3 p.S9G polymorphism in the development of ICDs in PD may be explained by this form of the receptor demonstrating a reduced binding affinity to dopamine [35], which may result in inappropriate dopamine-mediated stimulation of the mesocorticolimbic pathway.

Imaging studies have revealed overactivity of the mesocorticolimbic pathway, which consists of the OFC, amygdala, hippocampus and insula [29–34]. The OFC, together with the amygdala, demonstrate a role in learning from negative events [18]. Impulsive behaviours in PD may, therefore, be explained by dysfunction in the OFC-amygdala circuit resulting in an inability to learn from negative outcomes due to OFC overactivity. Overactivity of the hippocampus, which is associated with novelty processing [18], and the insular cortex, which is important in decision making [18], along with the OFC and amygdala may provide a pathological basis for the development of ICDs in PD. Involvement of the STN may add to this pathological basis, as shown by the presence of impulsive behaviour after STN-DBS in patients with PD [37–39]. This may be explained by possible indirect activation of the OFC via the hyper-direct pathway [19,20] or disinhibition induced by DBS [21,22].

### 4.1. Limitations

This review has methodological limitations. Unpublished studies have not been included, thereby limiting acknowledgment of the most up-to-date evidence. This review is also restricted by limitations of the studies themselves. Recruitment of patients in all studies occurred within a clinical setting, predominantly at specialised clinics, therefore providing a biased insight into impulsive behaviours in patients with PD. ICDs can resemble obsessive-compulsive disorders, making it difficult to distinguish ICDs as a homogenous group [23]. It is also important to consider interviewer and responder bias in cases where questionnaires have been used given the stigma surrounding the topic of impulsive behaviours. In the case of imaging studies, the activity of certain brain regions does not necessarily signify a direct association of this area with a particular process e.g. gambling. Evidence derived from STN-DBS needs to take into account the possibility of lesioning effects of the STN, altered task performance from reduced motivation and the influence of pre- and post-operative management with DAs.

### 4.2. Clinical implications

Patients and their carers should be informed of the possibility of developing ICDs given its negative influences on quality of life. Patients may be screened using the validated Questionnaire for Impulsive-Compulsive Disorders in PD (QUIP) [2]. Patients and carers need to be educated and warned of the development of pathological gambling, hypersexuality and other such impulsive behaviours. Clinicians should assess the likelihood of a patient developing ICDs by using QUIP and should prompt the discussion of such behaviour with appropriate adjustment of medication accordingly, involving carers where possible. Surgical intervention needs to be carefully considered given the limited evidence.

### 4.3. Future research

Research is required to further investigate the role of medication used in the management of PD, particularly DAs, in the development of ICDs. Randomised controlled trials are required to provide a better hierarchy of evidence and a greater number of prospective studies are required in order to better establish potential causative factors. Given the limited evidence of the effects of STN-DBS, further research is required in this area. It may also be important to distinguish the different behaviours in ICDs e.g. compulsive shopping, pathological gambling and hypersexuality and determine their pathophysiology in PD in order to find better identify potential treatment methods.

## 5. Conclusion

Our systematic review of the recent research on the pathophysiology of ICDs in PD suggests that both intrinsic and extrinsic factors can be involved in the development of ICDs in patients with PD. Genetic polymorphisms together with dysfunction of the mesocorticolimbic pathway and STN may contribute to the development of impulsive behaviours in a predisposed individual. ICDs may also be induced by medications used in the management of PD, particularly DAs. Further evidence is required to determine the exact pathophysiology of ICDs in PD in order to inform the clinical management of these debilitating behaviours in the context of a neurodegenerative condition which affects patients' health-related quality of life in multiple ways.
